# The validity and reliability of the self-directed learning instrument (SDLI) in mainland Chinese nursing students

**DOI:** 10.1186/1472-6920-14-108

**Published:** 2014-05-27

**Authors:** Wang-qin Shen, Hong-lin Chen, Yan Hu

**Affiliations:** 1 Principal Lecturer in Nursing, School of Nursing, Nantong University, Nantong, Jiangsu, PR China; 2 Professor in Nursing, School of Nursing, Fudan university, Shanghai, PR China

**Keywords:** Self-directed learning, Nursing students, Scale, Validity, Reliability

## Abstract

**Background:**

Self-directed learning is crucial to the professional development of nursing students, and which enables them to expand the knowledge and enhance the quality of their practice. A validated self-directed learning instrument is important not only in assessing the individual’s self-directed learning level, but also in evaluating the effectiveness of teaching or learning methods. The aim of this study is to evaluate the validity and reliability of the SDLI in mainland Chinese nursing students.

**Methods:**

A cross-sectional design with convenience sampling was used to recruit participants from three nursing schools. The mainland Chinese version of SDLI was tested with respect to validity and reliability in 1,499 nursing students, and another 30 nursing students were invited to evaluate the test-retest reliability of the scale in 7 days interval.

**Results:**

Explorary factor analysis identified a four-factor structure, accounting for 56.101% of the total variance. The confirmatory factor analysis showed a good overall fit of this four-factor model. Convergent validity was supported by the highly positive Pearson’s correlation between SDLI score and SRSSDL score (r = .876, *p* = .000). Cronbach’s alpha for internal consistency of overall scale was .916, and 4 dimensions were between .755-.825.The test-retest reliability of overall scale was .850, and 4 dimensions were between .708-.821. The intraclass correlation coefficient (ICC) of overall scale was .916, and 4 dimensions were .822-.889.

**Conclusions:**

This study indicates that the SDLI is a valid and reliable instrument for assessing self-directed learning in mainland Chinese nursing students. Nurse educators could use such knowledge to develop their roles and plan to support nursing students in becoming self-directed learners and lifelong learner.

## Background

Self-directed learning (SDL) has been identified as an approach to learning that received increasing attention in recent years, particularly in the context of higher education [1]. Self-directed learning has been shown to be associated with increased curiosity, critical thinking, quality of understanding, retention and recall, better decision making, achievement satisfaction, motivation, competence and confidence [2-5]. It is a popular approach for learning in nursing education as it provides a valuable approach with regards to demands of the nursing profession.

Self-directed learning is of great importance to the professional development of nursing students, and which enables them to expand the knowledge and enhance the quality of their practice. Nursing students must keep abreast of new information, current and emerging trends, medical technology and related scientific and professional publications to be able to function effectively in a constantly changing workplace [6]. Self-directed learning helps nursing students remain flexible, open to change, current in practice skills, and at the same time it helps in the growth of the students’ confidence and professionalism [7]. In the nursing programs for both undergraduates and postgraduates, SDL has been widely used in the form of learning contracts, problem-based package and distance learning packages. Consequently, it is critical for nurse educators to realize the importance of supporting nursing students to direct their own learning successfully and in being a good facilitator.

Knowles defined SDL as a process in which individuals take the initiative, with or without the help of others, to diagnose their learning needs, formulate learning goals, identify human and material resources for learning, choosing and implementing appropriate learning strategies, and evaluating learning outcomes [8]. Garrison’s model of self-directed learning stated that the essential components of SDL were self-management, self-monitoring, and motivation [9]. Communication is a lifelong learning process for the nurse. Nurses make the intimate journey with clients and their families. Therefore, communication is essential for nursing education.

SF-Cheng developed and validated an instrument called “Self-Directed Learning Instrument (SDLI) for Nursing Students” to assess nursing students’ SDL abilities. Altogether, 20 self-directed learning items for nursing students were identified and classified into four domains: learning motivation, planning and implementing, self-monitoring, and interpersonal communication. These four domains matched the empirical evidences with the theory [10]. Both nursing educators and nursing students can benefit from the SDLI instrument. With SDLI, the educators can easily identify their students’ SDL level and their learning deficits as well, and can function better in guiding their students from dependent learning to self learning, with the full consideration of the different needs of each individual student. By responding to the SDLI items, the students cannot only discover their own SDL abilities, but also develop an insight into SDL and a better understanding of the concept, which is crucial for the development of their self-directed learning, independent and lifelong learning.

The validity and reliability of the SDLI was measured in 1,072 Taiwanese nursing students. The results show SDLI was a valid and reliable instrument for identifying students’ SDL abilities [10]. The SDLI was developed only by using confirmatory factor analysis, not testing its construct validity by exploratory factor analysis. In addition, personality traits and interpersonal relations in the demographic variables of nursing students, as well as curricular and teaching methods, were significantly different from each other in Taiwan and mainland China. Therefore, the general aim of this paper is to testify the validity and reliability of SDLI in mainland Chinese nursing students.

## Methods

### Participants

A sample of 1,499 nursing students of associate and baccalaureate degree programs from three schools of nursing in Shanghai and Jiangsu province participated in the study. A separate sample of 30 nursing students was chosen to establish the test-retest reliability with a 7-day interval between the first assessment and the second. This group was not included in the 1,499 nursing students mentioned above. All subjects were full time students.

### Instruments

*Self-Directed Learning Instrument (SDLI) for nursing students*[10]: Self-directed Learning Instrument (SDLI) is a self-report instrument that was specially developed to measure self-directed learning of nursing students. The SDLI contains 20 items across the four domains of learning motivation (LM, 6 items), planning and implementing (PI, 6 items), self-monitoring (SM, 4 items), and interpersonal communication (IC, 4 items). Learning motivation is defined as the inner drive of the learner as well as external stimuli motivating one to learn and to take responsibility for one’s learning. Planning and implementing is defined as the ability to independently set learning objectives, using appropriate learning strategies and resources in order to effectively achieve learning goals. Self-monitoring is defined as the ability to evaluate one’s learning process and outcomes. Interpersonal communication is defined as the ability of learners to interact with others to promote their own learning. All items of SDLI are positively stated. The respondent is asked to rate each item on a 5-point Likert scale ranging from 1 for “strongly disagree” to 5 for “strongly agree”. Thus, the total possible score on the SDLI ranges from 20 to 100. A higher score indicates a higher level of SDL.

*Self-Rating Scale of Self-Directed Learning(SRSSDL)*[11]: The SRSSDL was a self-evaluation tool on self-directed learning developed by Williamson SN and validated in 2007 in a group of 30 bachelor nursing students attending their first and their last year at Thames Valley University. SRSSDL is composed of 60 items articulated in five subscales: Awareness (12 items), Learning strategies (12 items), Learning activities (12 items), Evaluation (12 items), Interpersonal skills (12 items). Responses for each item are rated by using a five-point Likert scale: 5 = always, 4 = often, 3 = sometimes, 2 = seldom, 1 = never. All items are positively stated, with higher total score showing a higher level of SDL. SRSSDL is found to be an effective tool for self-assessment of SDL both for nursing students, nurses, and Radiologist technicians [12-15]. Therefore, the SRSSDL was used to evaluate the concurrent validity of SDL on mainland Chinese nursing students.

*Demographic questionnaire*: A demographic information sheet is used to acquire basic information, such as gender, age, level of education, school, and grade.

### Procedures

An agreement and ethical approval from three schools (①School of nursing, Fudan University ②School of nursing, Nantong University③Department of nursing, Xinlin College, Nantong University) before conducting the pilot study was obtained. The students were informed that the purpose of the study was to collect normative data for a new assessment measure with the purpose of testing the reliability and validity of that measure. Students were told that their participation was voluntary, that non-participation would not affect their academic results or future study, and that all information would be confidential. Students who agreed to participate in the study were asked to sign a consent form and were given three self-report scales as well as the brief demographic questionnaire together in a packet. Data were collected by taking class as a unit, the uniform instruction was used in the test without recording name, and the questionnaires were collected on the spot.

### Statistical analysis

Statistical analysis was undertaken using the Statistical Package for the Social Sciences (SPSS), version 15.0. Descriptive statistics, including the number and proportion were used to describe the study participants.

The validity and reliability of the SDLI were evaluated as follows: Construct validity was established by exploratory and confirmatory factor analysis. The entire study sample (n = 1,499) was divided into two halves randomly. The factor structure of the SDLI was first examined by exploratory principal component analysis with Varimax rotation in one investigation survey (n = 750). This exploratory method was not used to validate the initial factor structure of the SDLI. To further corroborate the stability of the factor structure, the other survey (n = 749) was analyzed by confirmatory principle analysis with oblique rotation. This method was chosen because the objective of this study was to validate the mainland Chinese version of SDLI scale, although a four-factor solution was known for the original Taiwan version. Concurrent validity examined by analyzing the Pearson’s correlation coefficients between SRSSDL and the mainland Chinese version of SDLI. The internal consistency was established by calculating Cronbach’s alpha coefficients and the intraclass correlation coefficient (ICC) [16]. The test-retest reliability was established to test the intra-rater reliability [17]. P-Values of < .05 were considered statistically significant and two-tailed.

## Results

### Demographic data (participant characteristics)

A total of 1,509 nursing students participated in the study and 1,499 (99.34%) questionnaires were completed for analysis. Participants were all full time students and entered college after graduating from senior high school. Their GPA ranged from 1.23 to 3.82 (M = 3.12 and SD = 0.79). The ages ranged from 18 to 24 years (M = 20.00 and SD = 1.18). Demographic data of all samples are summarized in Table 1.

**Table 1:** Demographic characteristics of the participants

**Variables**	**Category**	**Number**	**%**
Gender	Female	1440	96.1
	Male	59	3.9
Age	≦20	1065	71.1
	≧21	434	28.9
Level of education	ADP	579	38.6
	BDP	920	61.4
Grade	Freshman	530	35.4
	Sophomore	509	33.9
	Junior	336	22.4
	Senior	124	8.3

*ADP* associate degree programs, *BDP* baccalaureate degree programs.

### Validity

#### Explorary factor analysis

A principle axis factor analysis with Promax Rotation was used to determine the underlying constructs of the scale because the correlation coefficients among the factors ranged from .525 to .714 [18]. The Kaiser-Meyer-Olkin measure of sampling adequacy was .943, which suggests that these data very suitable for factor analysis, as the relationship between the variables of the question items were marvelous [19]. Bartlett’s test of sphericity reached statistical significance (P = .000), which supported the factorability of the correlation matrix.

Four factors extracted in the exploratory sample (n = 749), the number of which was determined by size of eigenvalues variance. Items with factor loading exceeding 0.40 and no cross loading were assigned to factors. Table 2 shows the factor loadings of each item. Based on previous studies .40 was used in the factor loading as the cut-off score for selecting the items to be retained in the scale [20,21]. Thus, items in this study that had a 0.40 or higher loading were retained. A four-factor solution was found to explain 56.101% of the total variance across all items. Factor 1, “learning motivation” accounted for 38.82% of the variance; Factor 2, “planning and implementing” accounted for 6.502% Factor 3, “self-monitoring” accounted for 6.082%; Factor 4, “interpersonal communication” accounted for 4.703%.

**Table 2:** Results of exploratory principle component analysis of the Mainland Chinese version SDLI (n = 749)

	**Factor 1**	**Factor 2**	**Factor 3**	**Factor 4**	**Name of factors**
1. I know what I need to learn.	.469				Learning motivation (LM)
2. Regardless of the results or effectiveness of my learning, I still like learning.	.645				
3. I strongly hope to constantly improve and excel in my learning.	.706				
4. My successes and failures inspire me to continue learning.	.715				
5. I enjoy finding answers to questions	.611				
6. I will not give up learning because I face some difficulties.	.672				
7. I can pro-actively establish my learning goals.		.653			Planning and implementing (PI)
8. I know what learning strategies are appropriate for me in reaching my learning goals.		.709			
9. I set the priorities of my learning.		.649			
10. Whether in the clinical practicum, classroom or on my own, I am able to follow my own plan of learning.		.760			
11. I am good at arranging and controlling my learning time.		.705			
12. I know how to find resources for my learning.		.494			
13. I can connect new knowledge with my own personal experiences.			.625		Self-monitoring (SM)
14. I understand the strengths and weakness of my learning.			.584		
15. I can monitor my learning progress.			.738		
16. I can evaluate on my own my learning outcomes.			.686		
17. My interaction with others helps me plan for further learning.				.532	Interpersonal communication (IC)
18. I would like to learn the language and culture of those whom I frequently interact with.				.597	
19. I am able to express messages effectively in oral presentations.				.715	
20. I am able to communicate messages effectively in writing.				.742	
Eigenvalue	7.763	1.300	1.216	0.941	
Percent total variance	38.814	6.502	6.082	4.703	

#### *Confirmatory principal component analysis*

**Figure 1:**
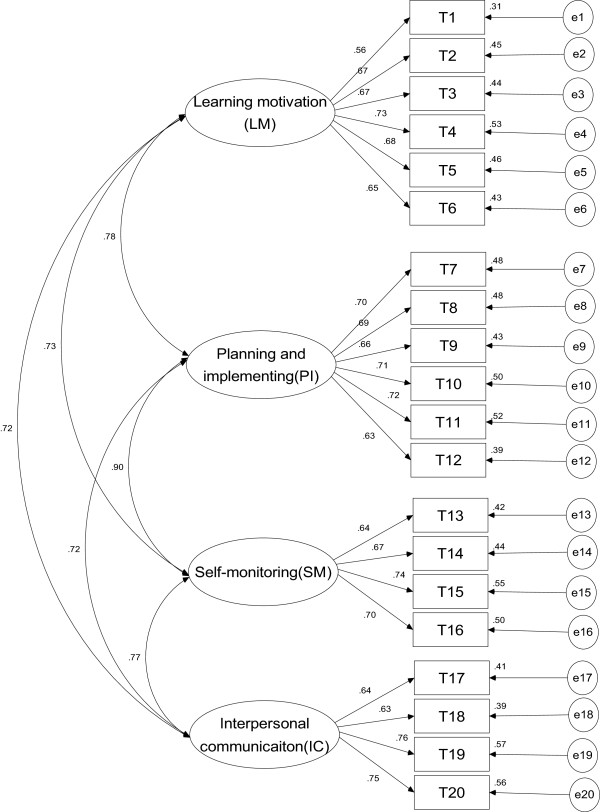
Confimatory factor analysis model of the SDLI.

As the two factors in exploratory analysis were intercorrelated (>.50), a confirmatory principal component analysis with oblique rotation was used for the other survey cases (n = 750) resulting in same four-factor structure (Table 2). The maximum likelihood factor analysis with oblique rotation was used to test the “goodness of fit” of the four-factor model (Figure 1). The results showed a good overall fit (RMR = 0.028, RMSEA = 0.057, CFI = 0.930, GFI = 0.929, AGFI = 0.909, PGFI = 0.781, NFI = 0.905) of the model. Therefore, a four-factor solution was found to be the most interpretable.

#### *Concurrent validity*

There was a good correlation between total score of SDLI and SRSSDL scores. The Pearson correlation between SDLI and SRSSDL was .876 (p = .000).

### Reliability

#### *Internal consistency reliability*

Reliability analyses showed good internal consistency for the SDLI (n = 1,499). Overall scale internal consistency of alpha was .916, and the four dimensions of Cronbach’s alpha were .813, .825, .759, and .755, respectively. The alpha score ranged from .482 to .743 if items were removed.

#### *The test-retest reliability*

The test-retest reliability was found to be high for SDLI total score (ICC was .916, P = .000), and four dimensions were also high, with an ICC range from .822 to .889 (p = .000, n = 30).

## Discussion

The aim of this study is to present the cross-cultural adaptation of the SDLI in a mainland Chinese population as well as to assess the psychometric potential of the mainland Chinese language version of the SDLI with regard to validity and reliability when compared to the SRSSDL. In total, 1,499 nursing students were evaluated. Good psychometric properties were found, proving that the SDLI scale were a valid and reliable instrument for assessing self-directed learning of nursing students at various grades.

The evidence for the construct validity of the mainland Chinese version of SDLI scale was supported by exploratory and confirmatory factor analysis. The exploratory principle axis factor analysis extracted four factors: “learning motivation,” “planning and implementing,” “self-monitoring” and “interpersonal communication,” accounting for 56.101% of the variance. In accordance with literature, SDL for nursing students is a multi-dimensional construct involving learning motivation, strategies or methods, activities, as well as interpersonal communication [11]. Of the four factors, the first factor “learning motivation”, which explains 38.814% of the total variable, should be considered as the most crucial dimension of an effective SDL. This factor seems to be higher than the first group of factor (25.756%) obtained in Italy version of SRSSDL by Cadorin L [14], and close to the “motivation” factor (34.379%) obtained in Chinese version of SRSSDL [13]. The factor is higher than Italy version of SRSSDL may be due to a higher amount of factors (eight) has emerged in the study by Cadorin L, and a high quantity of factors allows a more detailed analysis of the SDL [14]. The second factor “Planning and implementing” explains 6.502% of the total variable. “Planning and implementing” includes learning methods, learning strategies and skills needed to effectively implement the process of SDL. The third factor “self-monitoring” explains 6.082% of the total variable. The second and the third factors explain the close percent of the variation in their solution. The fourth factor “interpersonal communication” explains 4.703% of the total variable, on the verge of the “interpersonal skills” factor defined in the study by Cadorin L [14].

Further confirmatory maximum likelihood method supported a good fit to the model, as indicated by the Root Mean Squared Error of Approximation (RMSEA), the root mean square residual (RMR), the Goodness of Fit Index (GFI), Adjusted Goodness of Fit Index (NFI), and the Akaike Information Criterion (AIC). The χ2 values for model remained significant, possibly due to the large sample size and small discrepancies. The values of RMSEA, RMA, GFI, AGFI, NNFI, and NFI supported the acceptable fit of the model. Results show that the mainland Chinese version of SDLI scale can be used to reflect the self-directed learning of nursing students and also the functioning in the four dimensions, similar to results presented by Su-Fen Cheng. The strong positive Pearson’s correlation between SDLI score and SRSSDL score (r = .867, p = .000. It demonstrated that concurrent validity was well.

In this study, the internal consistency of the mainland Chinese version of SDLI scale and its 4 dimensions, as measured by Cronbach’s alpha, were all greater than .700. They were highly satisfactory [22]. The internal consistency were consistent with those of the original study [10].

ICC was the most appropriate and reliable parameter for repeated measurement on a continuous scale [23]. Test-retest reliability of the whole scale and its four dimensions was good (ICC, .822- .916). Those indicated that there were acceptable evidence of internal consistency tested by Cronbach’s alpha and test-retest reliability.

Because the SDLI was concise, with only 20 very straightforward items, most of the students in this study were able to complete it in about 5 minutes, with very few missing data points. The SDLI was proved to be manageable.

There are several limitations affected this study. The halo effect was a limitation of the study due to the use of the self-report questionnaire [24,25]. Future studies may use SDLI and other objective instruments to explore factors associated with SDL in nursing students, which enable nursing educators to understand how SDL works in mainland Chinese population.

## Conclusions

SDL is the key factor affecting lifelong learning abilities. Self-directed learning has been a part of basic learning training in many nursing programmes to prepare students to be lifelong learners. The feasible and effective tool evaluating the level of SDL is crucial in the self-directed leaning process for both nurse educators and nursing students. The findings of this study supported that SDLI is a valid and reliable instrument measuring the SDL level of nursing students. Additionally, the SDLI scale, which was composed of 20 items, is easy-to-use. Nurse educators can use SLDI to understand students’ SDL abilities better and implement appropriate teaching strategies to foster effective learning, and further the impact on teaching strategies. On the other side, the nursing students can use the SDLI to identify learning obstacles and seek relevant counseling and support.

## Competing interests

The authors declare that they have no competing interest.

## Authors’ contributions

SWQ made contributions to conception, study design, acquisition of data, data analysis, and in drafting the manuscript. CHL made contributions to conception and revision of the manuscript. HY made contributions to conception, study design, and in drafting the manuscript. All authors read and approved the final manuscript.

## Pre-publication history

The pre-publication history for this paper can be accessed here:

http://www.biomedcentral.com/1472-6920/14/108/prepub
